# Therapeutic Potential of Combined 5% Lifitegrast and Tocopherol Eye Drops in Managing Inflammation and Oxidative Stress in Murine Dry Eye

**DOI:** 10.3390/ph18010038

**Published:** 2025-01-01

**Authors:** Jayoung Moon, Enying Jiang, Jingting Liu, Hui Jin, Hee Su Yoon, Hoon-In Choi, Ji Suk Choi, Hong Qi, Hyeon-Jeong Yoon, Kyung Chul Yoon

**Affiliations:** 1 Department of Ophthalmology, Chonnam National University Medical School and Hospital, Gwangju 61469, Republic of Korealjt20001127@gmail.com (J.L.); gmltn0027@naver.com (H.S.Y.); hoonin_c@hanmail.net (H.-I.C.); kkangirri@hanmail.net (J.S.C.); yoonhyeonjeong@daum.net (H.-J.Y.); 2 Department of Ophthalmology, Peking University Third Hospital, Beijing 100191, China; doctorqihong@hotmail.com; 3 Beijing Key Laboratory of Restoration of Damaged Ocular Nerve, Beijing 100191, China

**Keywords:** lifitegrast, tocopherol, dry eye topical administration, ocular surface

## Abstract

**Background/Objectives**: This study aimed to evaluate the therapeutic effects of combined 5% lifitegrast (LF) and tocopherol (TCP) eye drops in a murine experimental dry eye (EDE) model. **Methods:** Female C57BL/6 were divided into seven groups: untreated controls, EDE control, EDE + 0.05% cyclosporin A (CsA), EDE + tocopherol (TCP), EDE + 5% LF, EDE + 5% LF + TCP (once daily), and EDE + 5% LF + TCP (twice daily). Clinical parameters (tear volume, tear break-up time (TBUT), corneal fluorescein staining score (CFSS), tear film lipid layer grade (TFLLG)) were assessed on days 7 and 14. Goblet cell density in the conjunctiva, CD4+ IFN-γ+ T cells, interleukin levels, reactive oxygen species (ROS) levels, and corneal apoptotic cells were analyzed on day 14. **Results:** Monotherapy with 0.05% CsA and LF showed improvements in all clinical parameters compared to the EDE control (*p* < 0.05). Combination therapy groups demonstrated superior improvements in clinical parameters compared to the EDE control, 0.05% CsA, and 5% LF groups. CD4+ IFN-γ+ T cell percentages and ROS levels in the cornea and conjunctiva were markedly reduced in the combination groups compared with the 0.05% CsA and 5% LF groups (*p* < 0.01). Furthermore, corneal apoptotic cells significantly decreased in the combination groups compared to the 0.05% CsA and TCP groups (*p* < 0.05). **Conclusions:** Combined 5% LF and TCP eye drops improved tear film parameters and reduced inflammatory and oxidative stress markers. The combination therapy can mitigate ocular surface damage by managing inflammation and oxidative stress in dry eye.

## 1. Introduction

Dry eye is a multifactorial disorder characterized by tear film instability, increased tear osmolarity, ocular surface inflammation, visual impairment, and discomfort, as outlined in the 2017 TFOS DEWS II report [1,2,3]. Elevated levels of inflammatory cytokines, chemokines, and immune response molecules in the tears and conjunctiva contribute to its pathogenesis, alongside oxidative stress, which damages the ocular surface and induces inflammation [4,5,6,7,8]. Suppressing inflammation and mitigating oxidative stress are therefore crucial for treating dry eye.

Conventional treatments, such as lubricants, topical cyclosporine (CsA), corticosteroids, tear-stimulating drugs, and autologous blood serum drops, focus on inflammation suppression but often result in slow relief of symptoms [9,10]. CsA ophthalmic solution (0.05%), an immunomodulatory agent approved by the FDA in 2003, reduces IL-2 production and T cell activation without directly targeting activated T cells [11]. While CsA can alleviate dryness, its effects may take several months to manifest [12]. Additionally, CsA often leads to a burning sensation, which has been a major limiting factor for its use in patients with dry eye.

In 2016, the FDA approved lifitegrast (LF), a novel anti-inflammatory agent for dry eye treatment. LF inhibits the interaction between intercellular adhesion molecule 1 (ICAM-1) and lymphocyte function-associated antigen 1 (LFA-1), targeting the inflammatory cycle central to dry eye [13]. LF has been shown to provide rapid symptom relief compared to CsA, with improvements observed as early as 2 weeks [13,14,15]. Still, LF cannot fully treat dry eye signs such as inferior corneal staining and has side effects such as eye irritation [15,16,17]. Surveys have revealed dissatisfaction among physicians with the effectiveness of current anti-inflammatory therapies, including lifitegrast, and high patient drop-out rates during treatment [16,17,18]. Although LF effectively inhibits T cell activation, it may not block oxidative stress, which exacerbates inflammation and causes cellular damage. Oxidative stress plays a critical role in amplifying inflammatory cascades, emphasizing the need for therapies targeting inflammation and oxidative damage [18,19]. To overcome these limitations, approaches targeting cellular damage and its underlying mechanisms, such as inflammation and oxidative stress, are crucial for improving therapeutic outcomes.

Tocopherol (TCP), a lipid with anti-oxidative and anti-inflammatory properties, can offer a promising solution to address both drug stability and oxidative stress-mediated inflammatory pathways central to dry eye pathogenesis. TCP neutralizes reactive oxygen species (ROS), specifically by interrupting free radical chain reactions and reducing oxidative degradation [20,21,22]. Additionally, it reduces pro-inflammatory cytokines and disrupts the inflammatory cycle [23,24]. As it targets oxidative stress-mediated inflammatory pathways, we assumed that TCP could enhance the therapeutic potential of LF and contribute to better clinical outcomes in dry eye.

In this study, we aimed to evaluate the therapeutic potential of a novel combination of lifitegrast and tocopherol eye drops in a murine dry eye model. Specifically, we assessed its impact on clinical parameters and goblet cell density, as well as biological markers, such as inflammatory cells and cytokines, ROS production, and corneal epithelial apoptosis. Additionally, we investigated whether once-daily dosing could provide comparable therapeutic benefits to twice-daily dosing.

## 2. Results

### 2.1. Clinical Parameters in the Tear Film and Ocular Surface

On day 7, the TCP, 5% LF + TCP[1], and 5% LF + TCP[2] groups showed a significant increase in the tear volume compared to the EDE group (all *p* < 0.01, Table 1). The 5% LF + TCP groups also showed an increase compared to the 0.05% CsA group (all *p* < 0.01), and the 5% LF + TCP[1] group showed an increase compared to the 5% LF group (*p* < 0.01). On day 14, all treatment groups showed a significant increase in tear volume compared to the EDE group (all *p* < 0.01). Furthermore, the 5% LF + TCP[1] and 5% LF + TCP[2] groups showed a significant increase in tear volume compared to the 0.05% CsA, TCP, and 5% LF groups (all *p* < 0.01).

The TBUT values at 7 days showed a significant increase in the TCP, 5% LF + TCP[1], and 5% LF + TCP[2] groups compared to the EDE group (all *p* < 0.01, Table 2). Both 5% LF + TCP[1] and 5% LF + TCP[2] groups showed a significant difference in TBUT compared to the 0.05% CsA and 5% LF groups at day 7 (all *p* < 0.01). At day 14, all treatment groups showed a significant increase in TBUT compared to the EDE group (all *p* < 0.01). The 5% LF + TCP[1] group showed a significant increase in TBUT compared to the 0.05% CsA and 5% LF groups (*p* = 0.02 and <0.01).

For CFSS at day 7, the TCP, 5% LF + TCP[1], and 5% LF + TCP[2] groups showed a significant increase in CFSS compared to the EDE group (all *p* < 0.01, Table 3). Additionally, the 5% LF + TCP[2] group showed a significant increase compared to the 5% LF group (*p* = 0.02). At day 14, all treatment groups exhibited significant improvements compared to the EDE group (all *p* < 0.01). Notably, both 5% LF + TCP[1] and [2] groups showed notable increases compared to the 0.05% CsA and 5% LF groups (*p* < 0.01 and 0.02 for 5% LF + TCP[1] and *p* < 0.01 and 0.02 for 5% LF + TCP[2], respectively).

### 2.2. Tear Film Lipid Layer Grades

**Figure 1:**
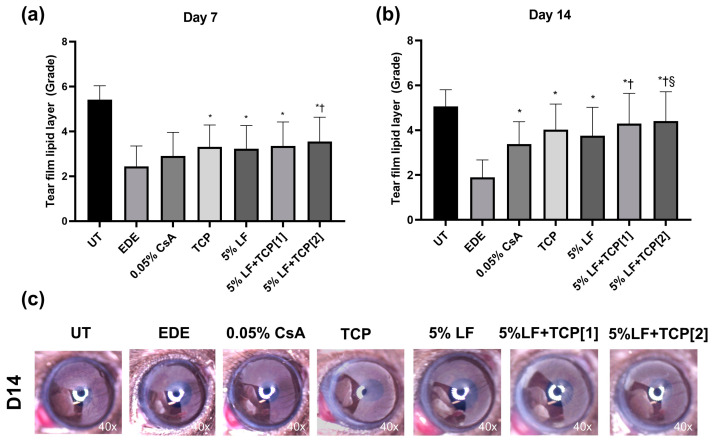
Mean tear film lipid layer grades at (**a**) day 7 and (**b**) day 14. (**c**) Representative figures in the untreated control (UT), experimental dry eye (EDE), 0.05% cyclosporin A (CsA), 0.01% tocopherol acetate (TCP), 5% lifitegrast (LF), 5% LF and TCP eye drop once-a-day [1], and 5% LF and TCP eye drop twice-a-day [2] groups on days 7 and 14. * *p* < 0.05 vs. EDE; † *p* < 0.05 vs. 0.05% CsA; § *p* < 0.05 vs. 5% LF.

Seven days after treatment, the mean TFLLGs were as follows: 5.41 ± 0.63 (UT), 2.44 ± 0.90 (EDE), 2.91 ± 1.05 (0.05% CsA), 3.31 ± 0.98 (TCP), 3.23 ± 1.03 (5% LF), 3.36 ± 1.06 (5% LF + TCP[1]), and 3.55 ± 1.09 (5% LF + TCP[2]). The TCP, 5% LF, 5% LF + TCP[1], and 5% LF + TCP[2] groups showed significant improvement compared with the EDE group (*p* = 0.02, 0.03, 0.04, and <0.01 respectively, Figure 1a). The 5% LF + TCP[2] group also showed better TFLLG than the 0.05% CsA group (*p* = 0.02).

After 14 days of treatment, the mean TFLLGs were 5.05 ± 0.75 (UT), 1.89 ± 0.78 (EDE), 3.38 ± 1.00 (0.05% CsA), 4.02 ± 1.14 (TCP), 3.75 ± 1.27 (5% LF), 4.29 ± 136 (5% LF + TCP[1]), and 4.41 ± 1.31 (5% LF + TCP[2]). All treatment groups showed a significant increase compared with the EDE group after 14 days of treatment (all *p* < 0.01, Figure 1b). Notably, both 5% LF + TCP groups exhibited a significant improvement compared with the 0.05% CsA group (all *p* < 0.01), while the 5% LF + TCP[2] group showed better results than the 5% LF group (*p* < 0.05). Representative images are presented in Figure 1c.

### 2.3. Conjunctival Goblet Cell Density

The mean goblet cell counts were 32.0 ± 1.4 cells/100 μm (UT), 7.0 ± 1.4 cells/100 μm (EDE), 12.0 ± 1.41 cells/100 μm (0.05% CsA), 17.0 ± 2.83 cells/100 μm (TCP), 21.0 ± 4.24 cells/100 μm (5% LF), 25.5 ± 3.54 cells/100 μm (5% LF + TCP[1]), and 25.5 ± 4.95 cells/100 μm (5% LF + TCP[2]). The 5% LF, 5% LF + TCP[1], and 5% LF + TCP[2] groups exhibited significantly higher conjunctival goblet cell densities than the EDE group (*p* = 0.03, <0.01, and <0.01, respectively). The goblet cell densities in both groups treated using 5% LF and TCP mixtures were significantly higher than that in the 0.05% CsA group (*p* = 0.03 and 0.02, respectively).

### 2.4. T Cell Activity and Inflammatory Cytokines in the Cornea and Conjunctiva

**Figure 2:**
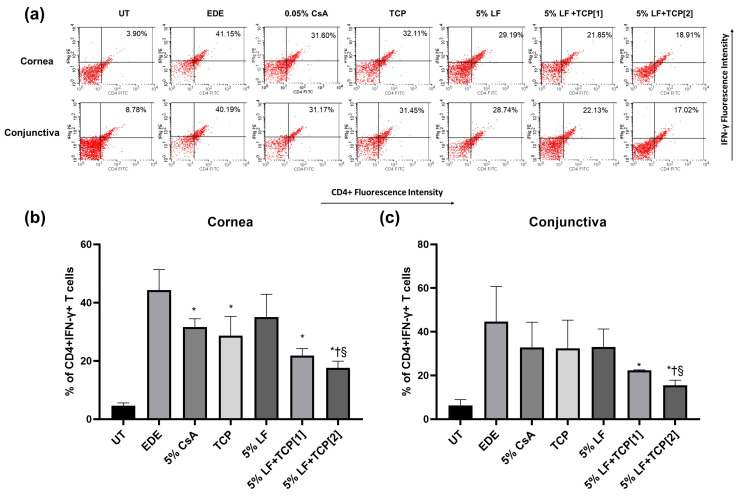
Flow cytometry showing CD4 + IFN-γ + T cells. (**a**) Representative images, and the mean percentages of CD4 + IFN-γ + T cells in the (**b**) cornea and (**c**) conjunctiva of the untreated control (UT), experimental dry eye (EDE), 0.05% cyclosporin A (CsA), 0.01% tocopherol acetate (TCP), 5% lifitegrast (LF), 5% LF and TCP eye drop once-a-day [1], and 5% LF and TCP eye drop twice-a-day [2] groups on day 14. * *p* < 0.05 vs. EDE; † *p* < 0.05 vs. 0.05% CsA; § *p* < 0.05 vs. 5% LF.

Figure 2a presents a representative histogram of CD4 + IFN-γ+ T cells in the cornea and conjunctiva, assessed via flow cytometry. The 5% CsA, TCP, 5% LF + TCP[1], and 5% LF + TCP[2] groups showed relatively lower percentages of corneal CD4 + IFN-γ+ T cells than the EDE group (all *p* < 0.05, Figure 2b). In addition, the 5% LF + TCP[2] group showed significantly lower percentages of corneal CD4 + IFN-γ+ T cells than the 0.05% CsA and 5% LF groups (*p* = 0.03 and <0.01). In the conjunctiva, both 5% LF + TCP groups showed a lower percentage of CD4 + IFN-γ+ T cells than the EDE group (*p* = 0.01, and <0.01, Figure 2c). The 5% LF + TCP[2] group showed a significant difference compared with the 0.05% CsA and 5% LF groups (both *p* = 0.04).

Inflammatory cytokine levels, specifically IL-1β and IL-6, were measured in the conjunctival tissues across all groups using Luminex assays (Table 4). IL-1β levels in the 5% LF + TCP[1] and [2] groups were significantly different compared to that in the EDE and TCP groups (all *p* < 0.01). IL-6 levels were significantly different between the 5% LF, 5% LF + TCP[1] and [2] groups and the EDE group (*p* = 0.02, <0.01, and <0.01, respectively). Furthermore, 5% LF + TCP[2] showed decreased IL-6 levels than 0.05% CsA group (*p* = 0.02).

### 2.5. ROS Levels in Corneal and Conjunctival Tissues

**Figure 3:**
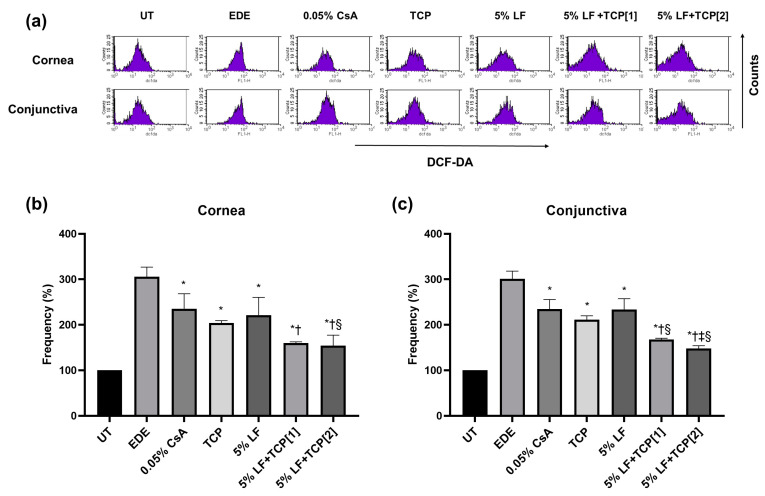
Relative frequency of extracellular reactive oxygen species measured using 2′,7′ dichlorodihydrofluorescein diacetate (DCF-DA). (**a**) Representative images and relative frequency of DCF-DA staining in the (**b**) cornea and (**c**) conjunctiva of the untreated control (UT), experimental dry eye (EDE), 0.05% cyclosporin A (0.05% CsA), 0.01% tocopherol acetate (TCP), 5% lifitegrast (LF), 5% LF and TCP eye drop once-a-day [1], and 5% LF and TCP eye drop twice-a-day [2] groups on days 7 and 14. * *p* < 0.05 vs. EDE; † *p* < 0.05 vs. 0.05% CsA; ‡ *p* < 0.05 vs. TCP; § *p* < 0.05 vs. 5% LF.

The representative samples of the intensity of ROS level measured using the DCF-DA assay are shown in Figure 3a. In the cornea, the EDE group exhibited a ROS level of 305.9 ± 20.86%, while the 0.05% CsA, TCP, and 5% LF groups showed levels of 235.5 ± 32.94%, 204.2 ± 5.11%, and 221.4 ± 38.86%, respectively, with the 5% LF + TCP[1] and 5% LF + TCP[2] groups further reduced to 160.1 ± 2.57% and 154.5 ± 22.78%, compared with the UT group (Figure 3b). In the conjunctiva, the EDE group exhibited a ROS level of 301.1 ± 17.00%, with the 0.05% CsA, TCP, and 5% LF groups showing levels of 234.7 ± 21.12%, 211.3 ± 8.54%, and 234.0 ± 23.64%, respectively, while the 5% LF + TCP[1] and 5% LF + TCP[2] groups showing levels of 167.9 ± 2.69% and 148.2 ± 5.89%, compared with the UT group (Figure 3c). Compared with the EDE group, all treatment groups represented a significantly lower intensity in the cornea and conjunctiva (all *p* < 0.05). The group with 5% LF and TCP mixtures showed a lower intensity than the 0.05% CsA group in the cornea and conjunctiva (all *p* < 0.05). The group with 5% LF and TCP twice a day showed better frequency in the cornea than the 5% LF group (*p* < 0.05). In the conjunctiva, the 5% LF + TCP[2] group showed decreased ROS levels compared with the TCP group (*p <* 0.01), and both 5% LF + TCP[1] and [2] groups showed stronger intensities than the 5% LF group (both *p* < 0.01).

### 2.6. Apoptotic Cells in the Corneal Epithelium

**Figure 4:**
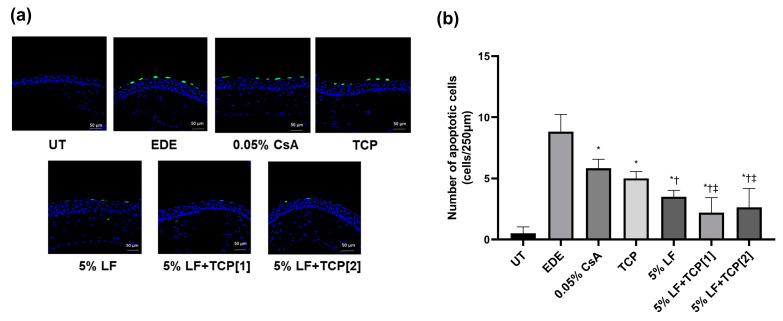
TUNEL assay of the cornea to identify apoptotic cells. (**a**) Representative specimens and (**b**) mean number of apoptotic cells in the untreated control (UT), experimental dry eye (EDE), 0.05% cyclosporin A (CsA), 0.01% tocopherol acetate (TCP), 5% lifitegrast (LF), 5% LF and TCP eye drop once-a-day [1], and 5% LF and TCP eye drop twice-a-day [2] groups on day 14. * *p* < 0.05 vs. EDE; † *p* < 0.05 vs. 0.05% CsA; ‡ *p* < 0.05 vs. TCP.

Representative images of corneal apoptotic cells from each group are shown in Figure 4a. The mean number of apoptotic cells was 0.50 ± 0.55 cells/250 μm (UT), 8.83 ± 1.47 cells/250 μm (EDE), 5.83 ± 0.75 cells/250 μm (0.05% CsA), 5.00 ± 0.58 cells/250 μm (TCP), 3.50 ± 0.55 cells/250 μm (5% LF), 2.20 ± 1.30 cells/250 μm (5% LF + TCP[1]), and 2.63 ± 1.60 cells/250 μm (5% LF + TCP[2]). All treatment groups demonstrated a significant reduction in apoptotic cells compared with the EDE group (all *p* < 0.01, Figure 4b). The 5% LF, 5% LF + TCP[1], and 5% LF + TCP[2] groups showed greater improvement than the 0.05% CsA group (all *p* < 0.01). Additionally, both 5% LF + TCP groups exhibited better results than the TCP group (all *p* < 0.01).

## 3. Discussion

CsA, one of the most widely used immunomodulatory agents for dry eye, suppresses inflammation but is limited by delayed therapeutic onset and side effects, such as burning sensations, which significantly impact long-term adherence [13,14,15,25]. 5% LF, an anti-inflammatory agent targeting ICAM-1 and LFA-1 interactions, has demonstrated a faster onset of action compared to CsA, as well as providing efficiency in reducing tear osmolarity and cytokine activity [26,27]. In the present study, both CsA and 5% LF monotherapy groups exhibited improvements in several clinical parameters including tear volume, TBUT, CFSS and TFLLG compared to the EDE group. However, these therapies may have limited efficacy in addressing oxidative stress, which plays a critical role in dry eye pathogenesis. To overcome these limitations, recent studies have explored therapeutic strategies combining agents with complementary mechanisms of action to enhance overall efficacy. For instance, antioxidants have been paired with anti-inflammatory agents to stabilize the tear film and improve ocular surface integrity [28,29,30]. We previously reported that the combination of diquafosol, a mucin secretagogue, and TCP improved tear film stability, reduced oxidative stress and inflammation, and enhanced conjunctival goblet cell density in a murine dry eye model [31].

This study demonstrated superior efficacy of 5% LF and TCP combined eye drops compared to monotherapy with 0.05% CsA, TCP or 5% LF in improving clinical parameters in an EDE model. Clinically, this combination therapy significantly improved tear volume and TBUT compared to the EDE control, CsA, TCP, and LF groups, while enhancing CFSS and TFLLG compared to the EDE and 0.05% CsA groups. The increased goblet cell density observed with combination therapy suggests enhanced mucin production, which is essential for tear film stability and ocular surface protection. The oil-based properties of TCP, a stabilized form of vitamin E, likely contribute to tear film enhancement by preventing lipid peroxidation and stabilizing the lipid layer [32,33,34]. This aligns with the observed reductions in corneal staining and improvements in TFLLG in the combination groups [31]. TCP likely contributes to this improvement by stabilizing the tear film lipid layer by its anti-oxidative properties, including the prevention of lipid peroxidation [35,36,37]. The combined eye drops may enhance multiple layers of the tear film, including lipid, mucin, as well as aqueous layers, thereby alleviating dry eye symptoms and supporting ocular surface integrity.

CD4 + IFN-γ+ T cells are known to decrease through the inhibition of T cell activation and migration by LF [27]. Flow cytometry revealed that a combination of 5% LF and TCP significantly reduced inflammatory CD4+ IFN-γ+ T cells in the cornea and conjunctiva compared to the EDE, 0.05% CsA, and 5% LF groups, indicating a more substantial modulation of inflammatory and oxidative pathways. Inflammatory cytokine levels, including those of IL-1β and IL-6, were also diminished in the 5% LF + TCP groups, compared to the 0.05% CsA, TCP and EDE groups.

The combination of 5% LF and TCP demonstrated further reductions in ROS levels in the cornea and conjunctiva compared to the 0.05% CsA and 5% LF groups. Additionally, the 5% LF + TCP groups demonstrated a greater reduction in corneal apoptotic cells than the 0.05% CsA and TCP groups, suggesting enhanced anti-oxidative and anti-apoptotic activity with the combination therapy. The enhanced reduction in ROS levels and significant decrease in corneal epithelial apoptosis suggests that the combination therapy may effectively interrupt oxidative stress-induced inflammation and cellular damage. These findings emphasize that TCP not only stabilizes LF’s anti-inflammatory effects but also alleviates oxidative stress-induced cellular damage, leading to comprehensive improvements in dry eye pathology [21,35,38,39]. Thus, the combined effects of TCP and LF offer a comprehensive therapeutic strategy for managing dry eye through complementary pathways while also improving clinical signs in patients with dry eye.

There was no significant difference between the once-daily (5% LF + TCP[1]) and twice-daily (5% LF + TCP[2]) applications of the combination eye drop, indicating non-inferiority of once-daily dosing (*p* > 0.05). This finding suggests that reducing the frequency of administration does not compromise therapeutic outcomes. Improved bioavailability and absorption of TCP, which enhances drug solubility and forms a stable lipid film on the ocular surface, likely contributed to the observed efficacy of the combination therapy [36,37,40]. Given the potential benefits of reduced application frequency—such as improved patient adherence, convenience, and reduced discomfort—further studies are warranted to confirm the optimal dosing regimen and its long-term efficacy in clinical settings.

This study has several limitations. First, the relatively short treatment duration limits our understanding of the long-term effects and durability of the observed benefits. Also, the small sample size and sensitivity of the assays may have restricted the ability to detect subtle changes. Future studies should focus on validating these findings through large-scale human clinical trials and exploring optimized dosing strategies, including the potential for once-daily dosing. Long-term studies are also needed to assess the durability of these benefits and their relevance across diverse patient populations with varying severities of dry eye.

## 4. Materials and Methods

### 4.1. Mouse Model of the Experimental Dry Eye and Experimental Design

This research protocol was approved by the Chonnam National University Medical School Research Institutional Animal Care and Use Committee (approval no. CNUHIACUC-23010). All procedures were conducted in compliance with the ARVO statement for the Use of Animals in Ophthalmic and Vision Research. Female C57BL/6 mice, aged 6 to 8 weeks, were used in this study. Mice were exposed to a dry environment for 18 h a day generated by a fan at 30% ambient humidity, as previously described [31,41]. Throughout the experiments, the animal’s behavior, as well as their food and water intake, were monitored without any restrictions.

**Figure 5:**
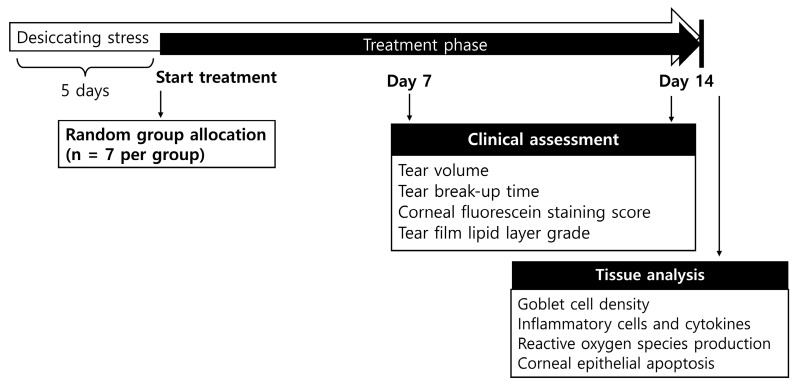
Experimental design for evaluating the therapeutic potential of combined 5% lifitegrast (LF) and tocopherol (TCP) eye drops. Mice were randomly allocated into seven groups (n = 7): Untreated without desiccating stress, experimental dry eye control (EDE) without any treatment, EDE + 0.05% Cyclosporine A, EDE + TCP, EDE + 5% LF, EDE + 5% LF + TCP once daily, and EDE + 5% LF + TCP twice daily.

After 5 days of desiccating stress, the mice were randomly assigned to seven groups based on the ophthalmic solution administered: untreated (UT) control mice (no desiccating stress or treatment); EDE control mice that received no ophthalmic solutions; EDE mice treated with 0.05% Cyclosporine A (0.05% CsA; Taejoon, Seoul, Korea) ophthalmic solution twice a day; EDE mice treated with 0.005% TCP acetate ophthalmic solution twice daily; EDE mice treated with 5% LF ophthalmic solution alone, twice daily; EDE mice treated with 5% LF and 0.005% TCP acetate mixture ophthalmic solution, once daily (5% LF + TCP[1]); and EDE mice treated with 5% LF and 0.005% TCP acetate mixture ophthalmic solution, twice daily (5% LF + TCP[2]). Each group consisted of seven mice and none were excluded during the experiments. A 2 μL-dose of the ophthalmic solution was applied topically to both eyes of the mice using a pipette. Tear volume, tear film break-up time (TBUT), corneal fluorescein staining score (CFSS), and tear film lipid layer grade (TFLLG) were measured in all animals (n = 14 eyes/group) at 7, and 14 days after treatment. Mice were sacrificed after measuring the clinical parameters on the 14th day post-treatment. PAS stain, flow cytometry, multiplex immunobead assay (Luminex), 2′, 7′-dichlorofluorescein diacetate (DCF-DA) assay, and TUNEL analysis were performed in 7 animals per group using tissue samples. The experiments were conducted in triplicate using seven distinct groups of mice. The study design and animal group allocation are detailed in Figure 5.

### 4.2. Evaluation of Tear Film and Ocular Surface Parameters

To assess tear film and ocular surface parameters, both eyes of animals (n = 14 per group) were evaluated on day 7 and 14. The tear volume was measured using phenol-red impregnated cotton threads (Zone-Quick, Showa Yakuhin Kako Co., Ltd., Tokyo, Japan) [31,42]. The length of the thread in contact with the outer canthus of the mouse for 20 s was measured using a microscope (SMZ 1500; Nikon, Melville, NY, USA) and converted into volume [43].

To assess TBUT, 1 μL of sodium fluorescein solution was instilled into the lower conjunctival sac. The time from lifting the eyelid until the tear film breaks was measured and recorded in seconds using a slit-lamp biomicroscope (BQ-900; Haag-Streit, Bern, Switzerland) with cobalt blue light. Ninety seconds after dye instillation, cornea epithelial damage was evaluated by a researcher blinded to the treatment groups.

The cornea was divided into four quadrants, and each was scored for CFSS based on a 4-point scale: 0, absent; 1, slightly punctuated staining (<30 spots); 2, punctate staining (>30 spots but not diffuse); 3, severe diffuse staining but no positive plaque; and 4, severe diffuse staining with positive fluorescein plaque [44]. The scores ranged from 0 to 16, summing up the scores in all four quadrants.

### 4.3. Evaluation of Tear Film Lipid Layer Grades

Both eyes of animals (n = 14 per group) were evaluated on day 7 and 14 to determine tear film lipid layer grades. A whiteboard was positioned at a specific location, and the light was reflected onto the lower half of the cornea. Following a blink, the researcher, who was blinded to the treatment conditions, used a trinocular stereo zoom microscope (SZM45TR-STL2, SOPTOP, Jinhua, China) to evaluate the interference pattern of the lipid layer on the corneal surface. The TFLLG was classified into six grades based on the lipid layer interference patterns as follows: Grade 1, grey appearance of low reflectivity and a faintly visible meshwork pattern; Grade 2, more compact meshwork pattern with a grey appearance of average reflectivity; Grade 3, vertical and horizontal grey waves of good visibility; Grade 4, yellow-grey and yellow spread lipid layer interference fringes superimposed on a background; Grade 5, brown-yellow and brown spread lipid layer interference fringes superimposed on a background; Grade 6, blue and brown spread lipid layer interference fringes superimposed on a background [45,46].

### 4.4. Evaluation of Conjunctival Goblet Cell Density

To assess conjunctival goblet cell density, animals were euthanized at day 14. Eyes and accessory organs were fixed in 4% paraformaldehyde, paraffin-embedded, and sectioned at 5-µm thickness. The sections were stained with Periodic Acid-Schiff (PAS) to assess goblet cell density, calculated as PAS-positive cells per 100 μm using light microscopy. For immunofluorescence, deparaffinized sections underwent antigen retrieval, blocking with 5% BSA, and overnight incubation with primary antibodies, followed by fluorescence-conjugated secondary antibodies. Nuclei were counterstained with DAPI, and stained sections were analyzed using a fluorescence microscope and image analysis software (Image-Pro version 10.0.5, Media Cybernetics, Silver Spring, MD, USA) to quantify inflammatory markers.

### 4.5. Measurement of T Cells and Inflammatory Cytokines

Corneal and conjunctival tissues were collected from each experimental group and processed to prepare single-cell suspensions for flow cytometric analysis. For immune cell analysis, cells were stained with fluorescence-conjugated antibodies specific for CD4 and IFN-γ to determine the count of CD4 + IFN-γ+ T cells. Flow cytometry was performed using the FACSCalibur cytometer (BD Biosciences, San Jose, CA, USA) with Cell Quest software (version 5.2.1; BD Biosciences) to quantify immune cell populations [47,48].

Conjunctival tissues from each group were also collected and homogenized for inflammatory cytokine analysis. Concentrations of cytokines such as TNF-α, interleukin-1β (IL-1β), and interleukin-6 (IL-6) were measured using a multiplex immunobead assay kit (Luminex 200; Luminex Crop., Austin, TX, USA) [31,47]. Tissue homogenates were incubated with antibody-coated magnetic beads in a 96-well plate for 2 h at room temperature. Following incubation, biotinylated detection antibodies and streptavidin-PE were added sequentially. The plate was then analyzed using an analysis system (xPONENT, Austin, TX, USA) to determine cytokine levels based on bead fluorescence.

### 4.6. Measurement of Intracellular ROS Level

To assess ROS levels, a CM-H2DCFDA kit was utilized [29]. Cells were incubated with DCF-DA dye (final concentration: 10 µM) for 30 min at 37 °C. The number of DCF-DA-positive cells was measured using the FACSCalibur cytometer at an excitation wavelength of 480 nm and an emission wavelength of 530 nm. The results were expressed as the mean percentage increase in DCF-DA fluorescence compared to that in the UT group using the CellQuest software version 5.2.1. 

### 4.7. Evaluation of Corneal Epithelial Apoptosis

To detect apoptotic cells in the cornea, a TUNEL assay was performed. Corneal tissue sections were treated with a TUNEL reaction mixture to label DNA strand breaks, a marker of apoptosis. The sections were then stained and observed under a fluorescence microscope, and the number of TUNEL-positive cells was counted to evaluate the extent of apoptosis in the corneal epithelium and stroma. TUNEL-positive cells and DAPI staining of cell nuclei were perceived under a microscope with 20-fold magnification.

### 4.8. Statistical Analysis

All data are presented as the mean ± standard deviation. Statistical analyses were performed using SPSS version 18.0 (SPSS, Inc., Chicago, IL, USA). The normality of data distribution was assessed using the Kolmogorov–Smirnov test. Differences in tear volume, TBUT, CFSS, and tear film lipid layer grade among the groups were analyzed using one-way repeated measures analysis of variance, followed by Dunnett’s post hoc tests. Sphericity was evaluated with Mauchly’s test, and if violated, adjustments were made using the Greenhouse–Geisser Epsilon correction. For other parameters such as flow cytometry values (CD4+ IFN-γ+ T cells), DCF-DA (ROS levels), cytokine levels (IL-1β, IL-6), conjunctival goblet cell density, and corneal apoptotic cells, the Kruskal–Wallis test with Bonferroni post hoc analysis was used to determine statistical differences between the groups. A *p*-value of less than 0.05 was considered statistically significant. Graphs were generated using GraphPad Prism version 9 (GraphPad Software, San Diego, CA, USA).

## 5. Conclusions

This study demonstrated that combined 5% LF and TCP eye drops significantly improved clinical and biological outcomes in a murine dry eye model. The combination therapy significantly improved tear volume, TBUT, CFSS, and TFLLG while increasing conjunctival goblet cell density and reducing oxidative stress (ROS levels), inflammatory T cells and cytokines, and apoptosis. Once-daily administration of the combination therapy provided comparable benefits to twice-daily dosing. These findings suggest that combined LF and TCP eye drops with anti-inflammatory and anti-oxidative mechanisms offer a promising therapeutic approach for dry eye disease, warranting further investigation in clinical settings.

## Figures and Tables

**Table 1:** Tear volume measurements at days 7 and 14 in murine dry eye model. Tear volume was measured in all groups, including untreated control (UT), experimental dry eye (EDE), 0.05% cyclosporin A (CsA), 0.01% tocopherol acetate (TCP), 5% lifitegrast (LF), 5% LF and TCP eye drop once-a-day [1], and 5% LF and TCP eye drop twice-a-day [2] groups. Data are presented as mean ± standard deviations.

Groups	Tear Volume at Day 7	Tear Volume at Day 14
UT	0.047 ± 0.009	0.044 ± 0.010
EDE	0.022 ± 0.008	0.019 ± 0.006
0.05% CsA	0.029 ± 0.012	0.030 ± 0.010 *
TCP	0.041 ± 0.017 *	0.033 ± 0.013 *
5% LF	0.034 ± 0.017	0.029 ± 0.011 *
5% LF + TCP[1]	0.052 ± 0.021 *^†§^	0.059 ± 0.016 *^†‡§^
5% LF + TCP[2]	0.048 ± 0.025 *^†^	0.051 ± 0.021 *^†‡§^

* *p* < 0.05 vs. EDE; ^†^ *p* < 0.05 vs. 0.05% CsA; ^‡^ *p* < 0.05 vs. TCP; ^§^ *p* < 0.05 vs. 5% LF.

**Table 2:** Tear break-up time measurements at days 7 and 14 in murine dry eye model. Tear break-up time was measured in all groups, including untreated control (UT), experimental dry eye (EDE), 0.05% cyclosporin A (CsA), 0.01% tocopherol acetate (TCP), 5% lifitegrast (LF), 5% LF and TCP eye drop once-a-day [1], and 5% LF and TCP eye drop twice-a-day [2] groups. Data are presented as mean ± standard deviations.

Groups	Tear Break-Up Time at Day 7	Tear Break-Up Time at Day 14
UT	2.49 ± 0.75	2.54 ± 0.56
EDE	1.32 ± 0.44	1.08 ± 0.50
0.05% CsA	1.51 ± 0.48	1.60 ± 0.50 *
TCP	1.75 ± 0.48 *	1.87 ± 0.47 *
5% LF	1.53 ± 0.43	1.56 ± 0.52 *
5% LF + TCP[1]	2.02 ± 0.44 *^†§^	2.00 ± 0.37 *^†§^
5% LF + TCP[2]	1.89 ± 0.56 *^†§^	1.84 ± 0.62 *

* *p* < 0.05 vs. EDE; ^†^ *p* < 0.05 vs. 0.05% CsA; ^§^ *p* < 0.05 vs. 5% LF.

**Table 3:** Corneal fluorescein staining score evaluated at days 7 and 14 in murine dry eye model. Corneal fluorescein staining score was measured in all groups, including untreated control (UT), experimental dry eye (EDE), 0.05% cyclosporin A (CsA), 0.01% tocopherol acetate (TCP), 5% lifitegrast (LF), 5% LF and TCP eye drop once-a-day [1], and 5% LF and TCP eye drop twice-a-day [2] groups. Data are presented as mean ± standard deviations.

Groups	Corneal Fluorescein Staining Score at Day 7	Corneal Fluorescein Staining Score at Day 14
UT	3.88 ± 1.73	4.50 ± 1.41
EDE	11.61 ± 1.74	13.23 ± 1.89
0.05% CsA	10.50 ± 2.15	10.57 ± 2.54 *
TCP	9.12 ± 2.33 *	9.64 ± 2.62 *
5% LF	10.88 ± 2.42	10.48 ± 1.90 *
5% LF + TCP[1]	8.79 ± 2.60 *	7.68 ± 2.13 *^†§^
5% LF + TCP[2]	8.76 ± 2.70 *^§^	8.48 ± 2.11 *^†§^

* *p* < 0.05 vs. EDE; ^†^ *p* < 0.05 vs. 0.05% CsA; ^§^ *p* < 0.05 vs. 5% LF.

**Table 4:** Inflammatory cytokine levels in the conjunctiva, measured by Luminex, for the untreated control (UT), experimental dry eye (EDE), 0.05% cyclosporin A (CsA), 0.01% tocopherol acetate (TCP), 5% lifitegrast (LF), 5% LF and TCP eye drop once-a-day [1], and 5% LF and TCP eye drop twice-a-day [2] groups. Data are expressed as mean ± standard deviations.

Groups	IL-1β (pg/mL)	IL-6 (pg/mL)
UT	24.23 ± 8.50	45.30 ± 10.32
EDE	61.60 ± 31.04	70.47 ± 16.03
0.05% CsA	48.90 ± 22.39	54.90 ± 17.29
TCP	50.90 ± 21.31	41.60 ± 15.98
5% LF	44.10 ± 22.80	44.33 ± 16.62 *
5% LF + TCP[1]	33.45 ± 20.19 *^‡^	35.53 ± 17.89 *
5% LF + TCP[2]	30.78 ± 18.39 *^‡^	25.30 ± 3.68 *^†^

* *p* < 0.05 vs. EDE; ^†^ *p* < 0.05 vs. 0.05% CsA; ^‡^ *p* < 0.05 vs. TCP.

## Data Availability

The datasets used and/or analyzed during the current study are available from the corresponding author upon reasonable request. The data are not publicly available due to [restrictions related to proprietary research methodologies].
